# Leukocyte Telomere Length Shortening Implies Plaque Instability and Major Adverse Cardiovascular Events in Patients With Angiographically Intermediate Lesions

**DOI:** 10.3389/fcvm.2021.812363

**Published:** 2022-01-21

**Authors:** Alian Zhang, Li Fan, Xiaolu Bao, Zuojun Xu, Zhaofang Yin, Yang Zhuo, Junfeng Zhang, Jun Gu, Alex C. Y. Chang, Yuqi Fan, Changqian Wang

**Affiliations:** ^1^Department of Cardiology, Shanghai Ninth People's Hospital, Shanghai JiaoTong University School of Medicine, Shanghai, China; ^2^Shanghai Institute of Precision Medicine, Shanghai Ninth People's Hospital, Shanghai JiaoTong University School of Medicine, Shanghai, China

**Keywords:** leukocyte telomere length, angiographically intermediate coronary lesion, thin-capped fibroatheromata, plaque instability, major adverse cardiovascular events (MACE)

## Abstract

**Background:**

Telomere shortening, an indicator of aging, is associated with age-related diseases. This study aims to investigate the association between leukocyte telomere length (LTL) and thin-capped fibroatheromata (TCFA) and the impact of using LTL cutoff to determine the incidence of major adverse cardiovascular events (MACEs) in patients with angiographically intermediate coronary lesions.

**Methods:**

This was a signal-center retrospective study focusing on patients who underwent coronary angiography and optical coherence tomography (OCT). The degree of coronary stenosis was assessed by angiography. The presence of TCFA was determined by OCT imaging. A total of 156 patients with angiographically intermediate coronary lesions were enrolled.

**Results:**

Leukocyte telomere lengths were significantly shorter in the TCFA group compared with non-TCFA group [11.95 (10.56, 15.21) kb vs. 13.81 (12.06, 16.11) kb, *p* = 0.003]. The short-LTL group and long-LTL group were divided according to the optimal cut-off value which was determined by the receiver operating characteristic (ROC) curve analysis. Logistic regression model revealed that short-LTL was independently associated with TCFA incidence (odds ratio [*OR*] 4.387, 95% *CI*: 1.902–10.120, *p* = 0.001) after adjusting for confounding factors. Over a 24-months follow-up, the MACE incidence among patients with short-LTL was significantly higher than those in the long-LTL group (12.5 vs. 2.0%, *p* = 0.006 by log-rank test). Multivariable cox regression analysis indicated that short-LTL (hazard ratio [*HR*] 9.716, 95% *CI*: 1.995–47.319, *p* = 0.005) was an independent prognostic factor of MACE incidence in angiographically intermediate coronary lesions patients.

**Conclusions:**

Short-LTL was independently associated with the incidence of TCFA and may serve as a prognostic factor for MACE risk on top of conventional risk factors.

## Introduction

Telomere, hexamer repetitive sequence, together with shelterin proteins form the protective capping complex at the end of eukaryotic chromosomes. Telomeres are considered to be the markers for cellular aging which gradually shorten during cell division (1). Telomere shortening is associated with many pathological processes, especially aging-related diseases, such as cancer, Parkinson's, and cardiovascular diseases (2, 3). Many epidemiological studies have suggested the association between leukocyte telomere length (LTL) and cardiovascular ischemic events (4–6). However, the relationship between LTL and coronary plaque instability has not been fully evaluated, especially in angiographically intermediate coronary lesions.

It has been reported that ~70% of acute coronary syndromes occur in previously angiographically intermediate coronary lesions (7) and about 6% of angiographically intermediate coronary lesions will worsen and require revascularization within 1 year (8). Although histology remains the gold standard for plaque classification, other imaging modalities have been assessed against histology to non-invasively identify plaque subtypes. Of these, optical coherence tomography (OCT) is currently the best validated against histology with excellent intra-observer reproducibility to identify plaque composition and fine structural changes in coronary arterial walls, such as thin-capped fibroatheromata (TCFA), macrophages, microvessels, and cholesterol crystals (9). TCFAs are thin fibrous caps that separate a necrotic core from the vascular lumen, which along with macrophage accumulation, microvessels, and cholesterol crystals, are considered as the characteristic of unstable plaques (10). Postmortem studies demonstrate that 60–70% of myocardial infractions (MI) are caused by the rupture of TCFA in patients without significant vessel stenosis (11).

As a result, the objective of this study was to investigate the correlation between LTL and TCFA measured by OCT and the predictor effect of short LTL on the incidence of major adverse cardiovascular events (MACEs) in patients with angiographically intermediate coronary lesions.

## Methods

### Study Design

A study sample was retrospectively selected from Shanghai Ninth People's Hospital, Shanghai Jiao Tong University School of Medicine, Shanghai, China during the period of January 2016 to September 2019. Patients with the angiographically intermediate coronary lesion (40–70% diameter stenosis) and further underwent OCT were enrolled. The exclusion criteria include (1) st-segment elevation myocardial infarction (STEMI) and non-st-segment elevation myocardial infarction (NSTEMI); (2) previous established coronary heart disease (CHD); (3) malignant tumor; (4) inflammation diseases; (5) patients with a declined renal function of estimated glomerular filtration rate (eGFR) ≤ 30 ml/min/1.73 m^2^ or who underwent hemodialysis; (6) severe hepatic dysfunction; (7) left main disease, heavily calcified vessels, or extremely tortuous; and (8) target lesion needs percutaneous coronary intervention (PCI).

**Figure 1:**
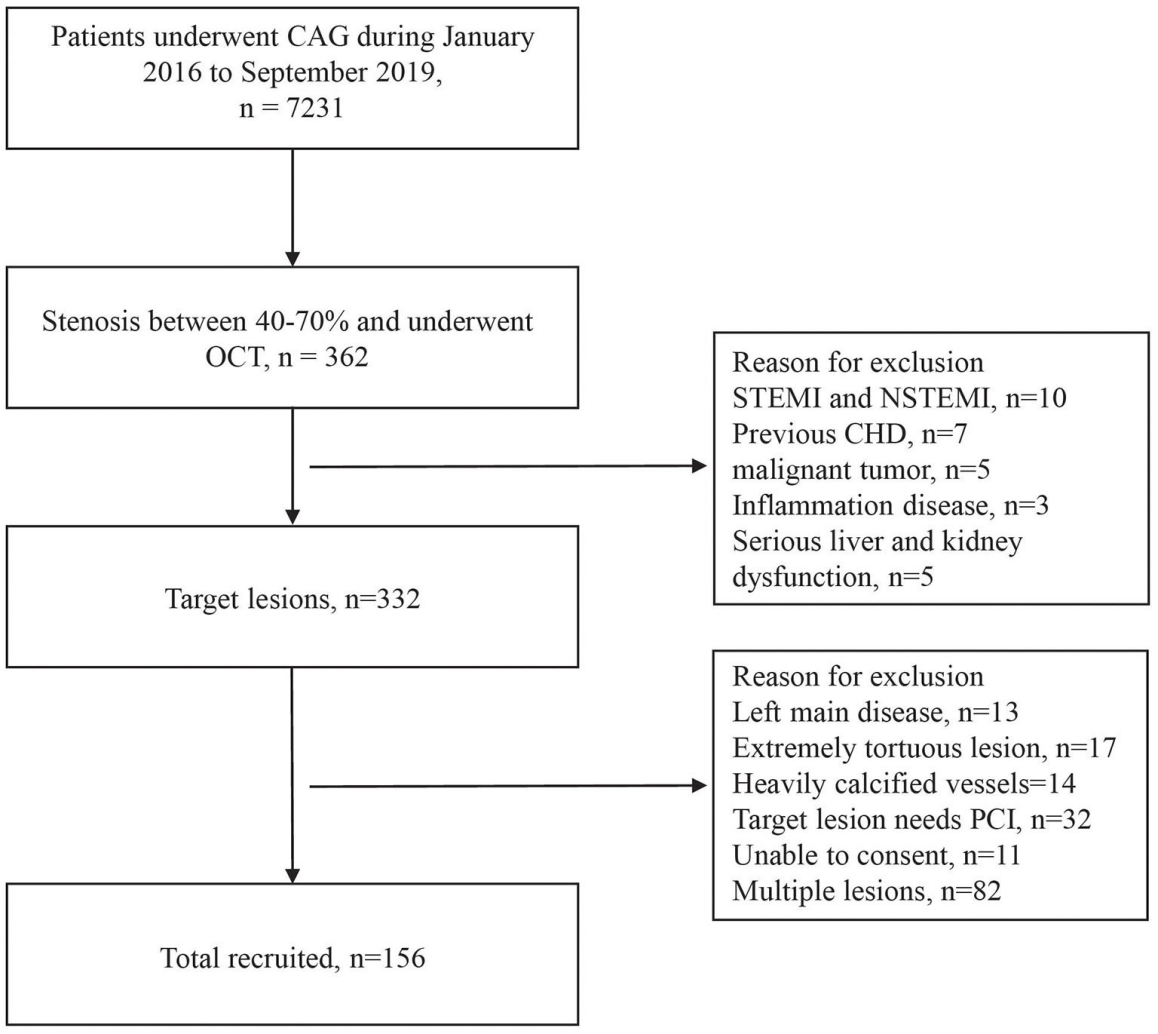
Inclusion and exclusion flowchart. CAG, coronary arteriography; OCT, optical coherence tomography; STEMI, st-segment elevation myocardial infarction; NSTEMI, non-st-segment elevation myocardial infarction; CHD, coronary heart disease; PCI, percutaneous coronary intervention.

In this study, 362 patients with angiographically intermediate coronary lesions and underwent OCT was included. On the basis of the exclusion criteria, 124 patients were excluded and 82 patients with multiple lesions were excluded. The final study sample comprised of 156 lesions from 156 patients (Figure 1). Participants were followed-up for MACEs up to September 2021.

Blood samples were collected in the morning after overnight fasting when hospitalized, including kidney function, glucose glycated hemoglobin (HbAlc), and lipid profiles. eGFR was calculated according to the CKDEPI equation. Blood cell samples for LTL measurement were stored at −80°C prior to DNA processing at the biobank of the Shanghai Ninth People's Hospital.

The study protocol was approved by the ethics committee of the Ninth People's Hospital, Shanghai Jiao Tong University School of Medicine. Procedures were performed in accordance with the Declaration of Helsinki and each patient gave written informed consent.

### Angiogram and Analysis

Coronary angiography was performed according to generally accepted routines. Quantitative coronary angiography (QCA) was performed using Cardiovascular Angiography Analysis System 5.10 by two independent doctors. The minimal luminal diameter (MLD) was defined as the smallest lumen diameter in the segment of a lesion. The reference vessel diameter (RVD) was calculated as the averaged diameter of the proximal and distal coronary segments without obvious narrowing. The diameter stenosis (DS) was calculated as follows:


(1) $$\begin{array}{r} D_{s}=\frac{\left(D_{r}-D_{m}\right)}{D_{r}}\times 100\% \end{array}$$

where *D*_*r*_ is RVD, *D*_*m*_ means MLD, and *D*_*s*_ represents DS.

### OCT Imaging and Analysis

Optical coherence tomography was conducted as described previously using a frequency-domain OCT imaging system (St. Jude Medical Inc.C7-XRT-MOCT, MN, USA). In brief, an OCT catheter (C7Dragonfly™) was sent to the distal site of the target lesion. The contrast medium was infused into the coronary artery by a high-pressure injector. The vessel was imaged with an automatic pullback device at 3 mm/s.

Optical coherence tomography image analysis was performed using offline Light Lab Imaging software in a blinded fashion by two experienced cardiologists individually, who were blinded to the information of patients. The morphology of the lesions was evaluated based on the typical Intravascular OCT guideline (2012) (12). In case of disagreement between the reviewers, a third reader was involved. Total 238 patients had 340 intermediate lesions. Of 238 patients, 156 patients had one intermediate lesion, 62 patients had two intermediate lesions, and 20 patients had three intermediate lesions. These patients with multiple lesions were excluded.

Thin-capped fibroatheromata (TCFA) was defined as a plaque with fibrous cap thickness <65 μm overlying a lipid-rich plaque (maximum lipid arc <90 degrees). Macrophage accumulation normally showed multiple strong back reflections that exceed the intensity of backscattering. Microvessels were defined as non-signal tubule luminal structures with a diameter of 50–300 μm and usually followed in at least three contiguous frames. Cholesterol crystals are characterized by the presence of linear and highly-reflecting structures within the plaque. The lipid arc was measured at 1 mm intervals throughout the length of each lesion and the values were averaged. The lipid length was measured as the consecutive longitudinal length of the plaque. The lipid index was calculated by multiplying the mean lipid arc by the lipid length (10, 12).

### Real-Time PCR Quantification of Telomere Length

DNA extraction was performed by using a commercial kit (Easy Pure Blood Genomic DNA Kit, Trans-Gen Biotech, Beijing, China) following standard procedures. The average LTL of patients' DNA samples were quantified by real-time quantitative PCR (RT-qPCR) in a LightCycler480 II Real-Time PCR system (Roche Diagnostics International, Rot-kreuz, Switzerland) using Scien-Cell's Absolute Human Telomere Length Quantification qPCR Assay Kit (Carlsbad, CA, USA) according to the protocol of manufacturers.

### MACEs Assessment

Major adverse cardiovascular events were recorded if the patient had any cardiovascular event within 24 months, such as death from cardiovascular causes, non-fatal myocardial infarction (MI), or an ischemia-driven target lesion revascularization (TLR) procedure. Follow-up data collection was performed based on a comprehensive medical record database, clinical visits, or phone call interviews.

### Statistical Analysis

All statistical analyses were performed using SPSS26 (SPSS, IBM, Armonk, NY, USA). Quantitative variables were expressed as mean ± SD for normal distribution data, while non-normal data were represented as medians and quartiles. Qualitative variables were expressed as numbers and percentages unless otherwise specified. For quantitative variables analysis, unpaired two-tail Student's *t*-test or the Mann–Whitney test were used according to the data distribution, with or without normality. A two-sided chi-square test was used to compare qualitative variables. The relationships between two numerical variables were investigated using simple linear regression analysis. Logistic regression analyses of relevant variables were performed to identify the risk factors for TCFA. The incidence of MACE was estimated by the Kaplan–Meier method, and any differences in MACEs were evaluated with a stratified log-rank test. A Cox proportional hazards regression model was used to estimate the effects of prognostic factors on MACEs. All predictors with a significance of *p* < 0.10 in the univariable analysis were entered into the multivariable model. A *p* < 0.05 was considered statistically significant.

## Results

### Baseline Clinical Characteristics

A total of 156 lesions of 156 patients (105 men and 51 women); mean age (65.29 ± 8.41 years) were included (TCFA group: *n* = 43, 27.6%; non-TCFA group: *n* = 113, 72.4%). The clinical characteristics were shown in Table 1. Compared with those in the non-TCFA group, patients in the TCFA group were more likely to be male (81.4 vs. 61.9%, *p* = 0.021) and have elevated TC levels (4.44 ± 1.26 mmol/L vs. 4.01 ± 1.01 mmol/L, *p* = 0.030). There were no significant differences in age, smoker status, CHD family history, hypertension, diabetes, and hyperlipidemia between these two groups. No significant differences in systolic blood pressure (SBP), diastolic blood pressure (DBP), triacylglycerol (TG), low-density lipoproteins-cholesterol (LDL-C), high-density lipoproteins-cholesterol (HDL-C), eGFR, and HbAlc, or use of drugs were observed between the two groups. Next, we examined whether patients with TCFA exhibited accelerated aging using LTL as a readout. LTLs were significantly shorter in the TCFA group compared with non-TCFA group [11.95 (10.56, 15.21) kb vs. 13.81 (12.06, 16.11) kb, *p* = 0.003] suggestive of accelerated aging.

**Table 1:** Baseline clinical characteristics findings.

	**Total ** **(*n =* 156)**	**Non-TCFA ** **(*n =* 113)**	**TCFA ** **(*n =* 43)**	***P*-value**
Age, years	65.29 ± 8.41	65.17 ± 8.34	65.63 ± 8.68	0.761
Male, *n* (%)	105 (67.3)	79 (61.9)	35 (81.4)	0.021
Smoker, *n* (%)	89 (57.1)	63 (55.8)	26 (60.5)	0.595
CHD family history, *n* (%)	90 (57.7)	65 (57.5)	25 (58.1)	0.944
Hypertension, *n* (%)	83 (53.2)	60 (53.1)	23 (53.5)	0.965
Diabetes, *n* (%)	48 (30.8)	30 (26.5)	18 (41.9)	0.064
Hyperlipidemia, *n* (%)	56 (35.9)	44 (38.9)	12 (27.9)	0.199
SBP, mmHg	134.31 ± 16.68	133.67 ± 16.44	136.00 ± 17.36	0.438
DBP, mmHg	79.99 ± 9.76	80.21 ± 9.82	79.40 ± 9.71	0.642
TC, mmol/L	4.13 ± 1.10	4.01 ± 1.01	4.44 ± 1.26	0.030
TG, mmol/L	1.62 ± 1.02	1.57 ± 1.03	1.73 ± 1.00	0.396
LDL-C, mmol/L	2.75 ± 1.04	2.73 ± 1.01	2.82 ± 1.05	0.631
HDL-C, mmol/L	1.11 ± 0.57	1.12 ± 0.52	1.11 ± 0.68	0.965
Glucose, mmol/L	6.21 ± 2.22	6.30 ± 2.30	5.97 ± 2.01	0.417
HbA1c, (%)	5.97 ± 1.00	5.99 ± 0.91	5.90 ± 1.22	0.590
eGFR	76.71 ± 19.03	76.33 ± 18.60	77.70 ± 20.31	0.690
**Treatment at discharge**				
Anti-platelet, *n* (%)	143 (91.7)	105 (92.9)	38 (88.4)	0.358
Statins, *n* (%)	145 (92.9)	104 (92.0)	41 (95.3)	0.470
β-blockers, *n* (%)	39 (25.0)	27 (23.9)	12 (27.9)	0.605
ACEI/ARB, *n* (%)	72 (46.2)	54 (47.8)	18 (41.9)	0.507
CCB, *n* (%)	50 (32.1)	73 (32.1)	13 (30.2)	0.764
Insulin, *n* (%)	20 (12.8)	14 (12.4)	6 (14.0)	0.794
LTL (kb)	13.51 (11.07, 15.49)	13.81 (12.06, 16.11)	11.95 (10.56, 15.21)	0.003

*Values were represented by mean ± SD or median and quartile (25%, 75%) and n (%)*.

*CHD, coronary heart disease; SBP, systolic blood pressure; DBP, diastolic blood pressure; TC, total cholesterol; TG, triacylglycerol; LDL-C, low-density lipoproteins-cholesterol; HDL-C, high-density lipoproteins-cholesterol; HbA1c, glycated hemoglobin; eGFR, estimated glomerular filtration rate; ACEI, angiotensin-converting enzyme inhibitors; ARB, angiotensin receptor blockers; CCB, calcium channel blocker; LTL, leukocyte telomere length*.

### Coronary Arteriography (CAG) and OCT Results

There were no significant differences in vessel location, MLD, RVD, DS, and MLA between TCFA vs. non-TCFA groups. However, macrophages infiltration (79.1 vs. 36.3%, *p* < 0.001), microvessels (55.8 vs. 30.1%, *p* = 0.003), cholesterol crystals (ChCs) (72.1 vs. 39.8%, *p* < 0.001), and calcium rich plaque (55.8 vs. 35.4%, *p* = 0.021) were increased in TCFA group compared with non-TCFA group. In addition, lesion length [22.9 (18.8, 32.6) vs. 19.8 (12.7, 26.5) mm, *p* = 0.007], the lipid length [19.9 (14.8, 24.0) vs. 13.0 (6.2, 21.4) mm, *p* < 0.001], the mean lipid arc [240.0 (220.0, 320.0) vs. 176.0 (91.5, 237.0) degrees, *p* < 0.001], and the lipid index [4,814.0 (3,286.4, 6,741.6) vs. 2,700.8 (1,025.9, 4,306.0) mm^*^°, *p* < 0.001] were greater and fibrous cap thickness (FCT) [56 (45.0, 62.0) vs. 92 (66.5, 120.0) μm, *p* < 0.001] was thinner in TCFA group compared with non-TCFA group (Table 2).

**Table 2:** Angiographic and optical coherence tomography (OCT) analysis.

	**Total ** **(*n =* 156)**	**Non-TCFA ** **(*n =* 113)**	**TCFA ** **(*n =* 43)**	***P*-value**
Vessel location				0.638
LAD, *n* (%)	100 (64.1)	74 (65.5)	26 (60.5)	
RCA, *n* (%)	43 (27.6)	31 (27.4)	12 (27.9)	
LCX, *n* (%)	13 (8.3)	8 (7.1)	5 (11.6)	
**Angiographic analysis**				
RVD, (mm)	3.31 ± 0.88	3.37 ± 0.90	3.15 ± 0.81	0.155
MLD, (mm)	1.40 ± 0.66	1.46 ± 0.69	1.25 ± 0.55	0.054
DS(%)	59.26 ± 11.26	58.46 ± 11.52	61.36 ± 10.37	0.151
Lesion length (mm)	20.7 (13.6, 28.3)	19.8 (12.7, 26.5)	22.9 (18.8, 32.6)	0.007
**Qualitative OCT analysis**				
Macrophages, *n* (%)	75 (48.1)	41 (36.3)	34 (79.1)	<0.001
Microvessels, *n* (%)	58 (37.2)	34 (30.1)	24 (55.8)	0.003
ChCs, *n* (%)	76 (48.7)	45 (39.8)	31 (72.1)	<0.001
Calcium rich plaque, *n* (%)	64 (41.0)	40 (35.4)	24 (55.8)	0.021
**Quantitative OCT analysis**				
Lipid length, (mm)	14.9 (9.1, 22.9)	13.0 (6,2, 21.4)	19.9 (14.8, 24.0)	<0.001
mean lipid arc, (°)	206.0 (132.5, 254.0)	176.0 (91.5, 237.0)	240.0 (220.0, 320.0)	<0.001
Lipid index, (mm*°)	3119.6 (1242.5, 5171.4)	2700.8 (1025.9, 4306.0)	4814.0 (3286.4, 6741.6)	<0.001
FCT, (μm)	72 (46.5, 103.5)	92 (66.5, 120.0)	56.0 (45.0, 62.0)	<0.001

*Values were represented by mean ± SD or median and quartile (25%, 75%) and n (%)*.

Lipid index = lipid length *mean lipid arc degrees.

*TCFA, thin-cap fibroatheroma; LAD, left anterior descending coronary artery; RCA, right coronary artery; LCX, left circumflex artery; RVD, reference vessel diameter; MLD, minimal lumen diameter; DS, diameter stenosis; ChCs, Cholesterol Crystals; FCT, fibrous cap thickness*.

In addition, the correlations between OCT findings and LTL were analyzed. LTL was inversely correlated with lipid length (*r* = −0.166, *p* = 0.039), lipid arc (*r* = −0.227, *p* = 0.004) and lipid index (*r* = −0.195, *p* = 0.015). Moreover, LTL was positively correlated with FCT (*r* = 0.159, *p* = 0.048).

### Receiver Operating Characteristic (ROC) Curve Analysis for LTL

To determine the sensitivity and specificity of LTL for predicting TCFA in patients with the angiographically intermediate coronary lesion, we performed ROC analysis (Supplement Figure 1). The area under the ROC curve (AUC) of LTL was 0.655 (95% *CI*: 0.560–0.750, *p* = 0.003). The optimal cut-off value for LTL was 12.23 kb, and the sensitivity and specificity were 74 and 61%, respectively (Supplementary Table 1).

To determine whether LTL cutoff alone can further delineate incidence of TCFA and predict MACEs, we divided our study population into two groups according to the optimal LTL cut-off value: long-LTL group (LTL > 12.23 kb) and short-LTL group (LTL <12.23 kb). There was an increased incidence of TCFA in the short-LTL group (46.4 vs. 17.0%, *p* < 0.001). There was no significant difference in gender, smoker, family history, or hypertension, hyperlipidemia, TG, LDL-C, HDL-C, eGFR, and HbAlc, or drug usage between the two groups. Compared with the long-LTL group, patients in the short-LTL group were older (67.45 ± 7.51 vs. 64.09 ± 8.68 mmol/L, *p* = 0.016), have more diabetes history (41.1 vs. 25.0%, *p* = 0.0037), and exhibited an increase in TC levels (4.33 ± 0.95 vs. 4.02 ± 1.17 mmol/L, *p* = 0.010). The incidence of macrophages (78.6 vs. 31.0%, *p* < 0.001) and microvessels (53.6 vs. 28.0%, *p* = 0.002) were significantly higher in short-LTL group than long-LTL group. Importantly, mean lipid arc [227.0 (172.5, 254.8) vs. 185.0 (98.8, 246.3) degrees, *p* = 0.026] was greater and FCT [58 (40.0,76.5) vs. 94.5 (58.0, 127.0) μm, *p* < 0.001] was thinner in short-LTL group compared with long-LTL group (Supplementary Tables 2, 3).

### Independent Risk Factors of TCFA

Possible influencing factors in patients with TCFA, such as age, family history, smoker, hypertension diabetes, total cholesterol, and LTL were assessed. In univariate analysis, male, diabetes, total cholesterol, and LTL (<12.23 kb) were significantly associated with TCFA. The *OR* of TCFA for short LTL was 4.231 [95% *CI*: 2.018–8.871, *p* < 0.001]. In addition, multivariable regression analysis also demonstrated short LTL was an independent risk factor of TCFA (*OR* 4.387, 95% *CI*: 1.902–10.120, *p* = 0.001) (Table 3).

**Table 3:** Univariate and multivariate logistic regression model for prediction of thin-cap fibroatheroma (TCFA).

**Variable**	**Univariate analysis OR (95% CI)**	***P-*value**	**Multivariate analysis OR (95% CI)**	***P*-value**
Male	2.687 (1.141–6.331)	0.024	3.522 (1.330–9.324)	0.011
Age	1.007 (0.965–1.050)	0.760	0.996 (0.944–1.050)	0.875
CHD family history	1.026 (0.504–2.089)	0.944	1.015 (0.433–2.379)	0.973
Smoker	1.214 (0.594–2.482)	0.595	1.267 (0.497–3.228)	0.620
Hypertension	1.016 (0.502–2.054)	0.965	1.326 (0.561–3.133)	0.520
Diabetes	1.992 (0.954–4.158)	0.066	1.968 (0.818–4.735)	0.131
Hyperlipidemia	0.607 (0.282–1.306)	0.202	0.582 (0.236–1.438)	0.241
Total cholesterol	1.409 (1.026–1.936)	0.034	1.296 (0.907–1.852)	0.155
LTL (<12.23kb)	4.231 (2.018–8.871)	<0.001	4.387 (1.902–10.120)	0.001

*CHD, coronary heart disease; LTL, leukocyte telomere length*.

### Follow-Up and MACEs Assessment

**Figure 2:**
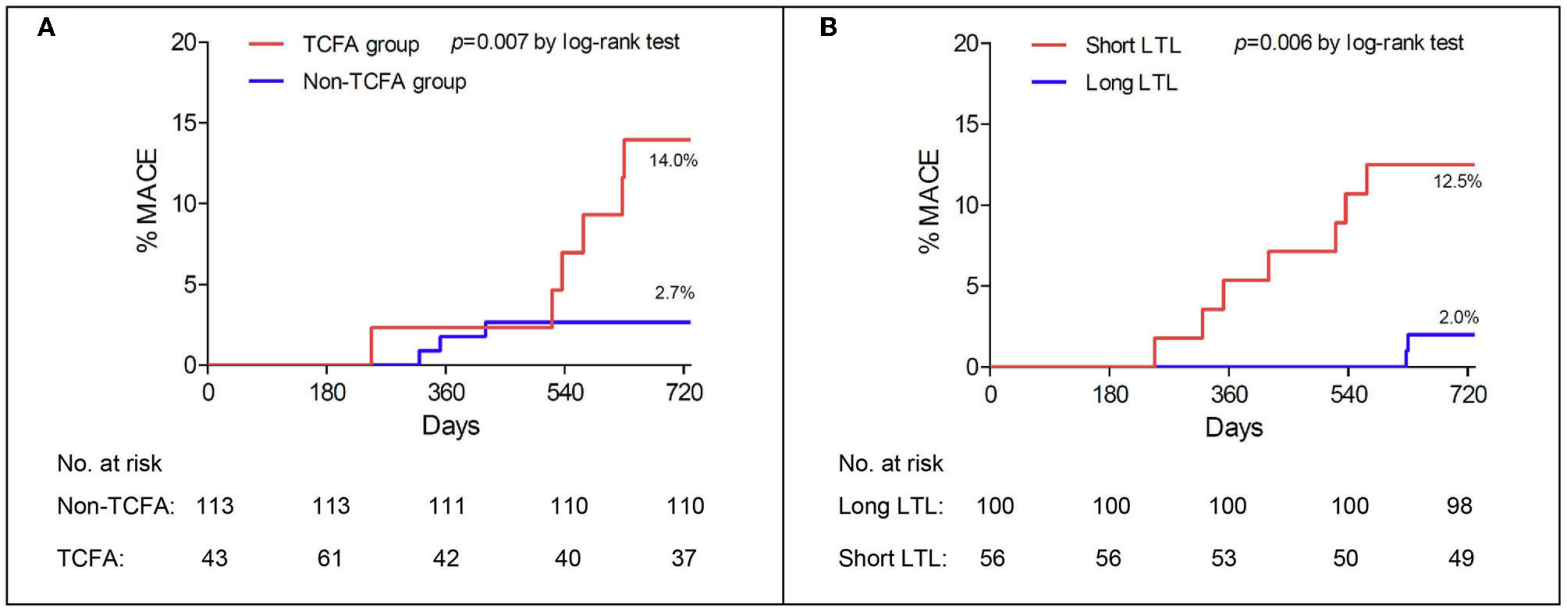
Major adverse cardiac events (MACEs) in the TCFA group and the non-TCFA group **(A)** and in the long LTL and the short LTL groups **(B)**. Results are expressed as Kaplan–Meier curves, and *p-*values are log-rank estimates. TCFA, thin-cap fibroatheroma; LTL, leukocyte telomere length.

After a follow-up of 24 months, 9 MACEs occurred. No deaths were reported. There were 6 events in the TCFA group and 3 MACEs in the non-TCFA group (14.0 vs. 2.7%, *p* = 0.007, by log-rank test) (Figure 2A). Incidence of MI (2, 4.7 vs. 0%, *p* = 0.021) and TLR (14.0 vs. 4.5%, *p* = 0.007) were higher in the TCFA group than the non-TCFA group.

Next, we examined if LTL can better segregate MACE incidence. MACE incidence was significantly higher in the short-LTL group than the long-LTL group (7, 12.5 vs. 2, 2.0%, *p* = 0.006 by log-rank test) (Figure 2B). One patient in the short LTL group and one patient in the long-LTL group (1, 1.8 vs. 1,1.0%, *p* = 1.000) suffered from acute MI. While ten patients in the short-LTL group and six patients in the long-LTL group (12.5 vs. 2.0%, *p* = 0.006) experienced TLR.

For multivariate regression analysis, variables with *p* < 0.10 (hyperlipidemia and LTL) from univariate regression analysis were entered into the multivariate cox proportional hazards model. It demonstrated that short-LTL (*HR* 9.716, 95% *CI*: 1.995–47.319, *p* = 0.005) was independently associated with an increased risk of MACE (Table 4).

**Table 4:** Multiple Cox analysis for the major adverse cardiovascular events.

**Variable**	**Univariate analysis HR (95% CI)**	***P*–value**	**Multivariate analysis HR (95% CI)**	***P–*value**
Male	0.958 (0.240–1.830)	0.951		
Age	0.991 (0.919–1.068)	0.807		
CHD family history	2.600 (0.540–12.515)	0.233		
Smoker	0.590 (0.159–2.199)	0.432		
Hypertension	1.806 (0.452–7.222)	0.403		
Diabetes	1.102 (0.276–4.405)	0.891		
Hyperlipidemia	6.629 (1.377–31.913)	0.018	9.598 (1.971–46.734)	0.005
Total cholesterol	1.096 (0.623–1.925)	0.751		
LTL (<12.23 kb)	6.720 (1.396–32.357)	0.018	9.716 (1.995–47.319)	0.005

*Hyperlipidemia and LTL were entered into the multivariate Cox proportional hazards model. In the multivariable adjusted models, all dependent variables were adjusted for each other*.

*CHD, coronary heart disease; LTL, leukocyte telomere length*.

## Discussion

In this study, we determined and compared the baseline characteristics between patients with TCFA and non-TCFA and found that TCFA patient LTLs were significantly shorter. Next, we sought out to show that LTL was an independent risk factor for TCFA and it was associated with an increased risk of MACE in patients with the angiographically intermediate coronary lesion. Importantly, LTL was independent of a variety of conventional risk factors.

Telomeres shorten with age, and aging is a major risk factor for CHD. Demanelis et al. measured the telomere lengths of different tissue types and found that LTL shortening is positively correlated with tissue aging, suggesting LTL can be used as a proxy for determining the biological age of a person (13). Existing epidemiologic studies have yielded conflicting results in terms of whether telomere lengths are correlated with CHD. Using Mendelian randomization, it has been demonstrated that a causal correlation exists between LTL shortness and increased ischemic heart disease risk independent of traditional risk factors (14). Mortality of heart disease in people with short LTL has been shown to be three times greater than people with long LTL in an elderly population (15). Samani et al. showed that LTL in patients with CHD was 303 base pairs shorter than health controls (16) and in a systematic review, shortened LTL is associated with a higher incidence of MI and cardiac death (6). However, contradicting findings have also been reported where LTL is not associated with CHD (angina or non-fatal ischemic heart disease) (6) or atherogenesis but is independently correlated with the presence of atherosclerotic plaques (17, 18). TCFA represents advanced atherogenesis and patients exhibiting this feature are of high risk. Here, we demonstrate that shortened LTL is an independent risk factor for TCFA. As far as we know, this is the first time to demonstrate the direct relationship between LTL and plaque instability in patients with the angiographically intermediate coronary lesion. It provides evidence that shortened LTL is involved in atherosclerosis progression.

Although the mechanism of how short telomeres promotes atherosclerosis remains unclear, there are several clues one may consider. Atherosclerosis by nature is an inflammatory disease that involves vascular smooth muscle, endothelial cells, and immune cells (19), and accumulation of senescent cells may further exacerbate this by reducing regenerative potential (20). It has been demonstrated that telomeres in coronary endothelial cells are shorter in atherosclerotic patients compared with healthy individuals (21); smooth muscle cells near plaque regions exhibit shorter telomeres than those near normal arteries (22). An increase in apoptosis and a decrease in proliferation of vascular smooth muscle cells result in fibrous caps thinning and plaque instability (23). Here, we show that LTL is significantly shorter in patients with TCFA compared with non-TCFA patients. Based on the available evidence, we speculate that aged circulation, short LTL, promotes atherosclerosis by inducing endothelial and vascular smooth muscle cell senescence which results in fibrous cap thinning.

Intermediate coronary lesion management remains a daily clinical challenge. Although OCT offers us the ability to assess coronary lesion that aids our treatment decision, the invasive nature and cost often make it prohibitive for mass scale implementation. Here, we show that patients with TCFA exhibit short LTL, and LTL is a selective risk factor for MACE incidence independent of conventional risk factors for patients with angiographically intermediate coronary lesions. Our results support the use of LTL as an alternative readout and combined with other risk factors, to provide superior risk stratification.

In our study, there are several limitations to be acknowledged. First, this study is a retrospective study with relatively small sample size. Second, different age groups were not analyzed in the study and the results of this study may be different in the other age groups. Third, patients who have coronary intermittent lesions but did not undergo an OCT examination were excluded, so selection bias may have occurred. Fourth, only one vessel imaging was performed. A prospective large-scale study is warranted and different age groups should be analyzed.

## Data Availability Statement

The raw data supporting the conclusions of this article will be made available by the authors, without undue reservation.

## Ethics Statement

The studies involving human participants were reviewed and approved by Ethics Committee of the Ninth People's Hospital, Shanghai Jiao Tong University School of Medicine. The patients/participants provided their written informed consent to participate in this study.

## Author Contributions

AZ, LF, XB, AC, and CW designed the experiments. LF, YZ, YF, ZY, and ZX performed coronary angiogram and OCT. LF and YF analyzed the OCT data. AZ and LF performed the experiments. AZ, XB, JG, and JZ analyzed the data. AZ, LF, XB, AC, and JZ wrote the manuscript. AZ, JG, AC, and CW edited the manuscript. All authors have read and approved the manuscript.

## Funding

This study was supported by research projects from the Shanghai Science and Technology Commission (18411950500 to CW); the SHIPM-mu fund from Shanghai Institute of Precision Medicine and the Ninth People's Hospital, Shanghai Jiao Tong University School of Medicine (jc201905 to AZ); the National Natural Science Foundation of China (82070248 to AC); and the project of construction and application of biobank for coronary heart disease of Shanghai Ninth People's Hospital (YBKA201910 to JZ).

## Conflict of Interest

The authors declare that the research was conducted in the absence of any commercial or financial relationships that could be construed as a potential conflict of interest.

## Publisher's Note

All claims expressed in this article are solely those of the authors and do not necessarily represent those of their affiliated organizations, or those of the publisher, the editors and the reviewers. Any product that may be evaluated in this article, or claim that may be made by its manufacturer, is not guaranteed or endorsed by the publisher.
