# Supplementary material for: Leukocyte Telomere Length Shortening Implies Plaque Instability and Major Adverse Cardiovascular Events in Patients With Angiographically Intermediate Lesions
Source: Front Cardiovasc Med. 2022 Jan 21;8:812363. doi: 10.3389/fcvm.2021.812363 (PMC8814518; doi:10.3389/fcvm.2021.812363)
Supplement: Supplementary file 1 [file Data_Sheet_1.docx]

Supplenment Figure 1. ROC Curve Analysis of LTL.


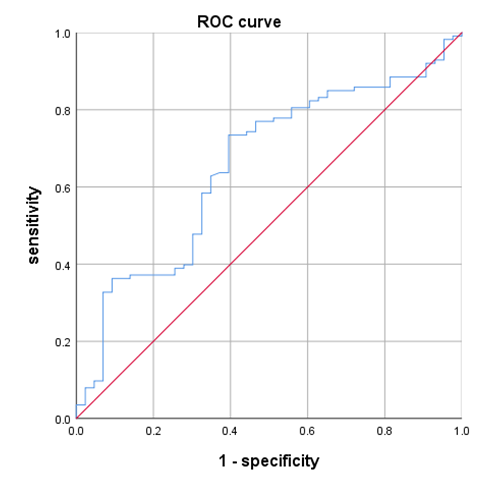


ROC, Receiver operating characteristic; LTL, leukocyte telomere length.

Supplenment Table 1. ROC curve for various cut-off levels of LTL.

| LTL (kb) | Specificity (95%CI) | Sensitivity (95%CI) | DOC |
| --- | --- | --- | --- |
| 11.97 | 0.535 (0.487-0.583) | 0.77 (0.722-0.818) | 0.519 |
| 12.23 | 0.605 (0.557-0.653) | 0.735 (0.687-0.783) | 0.476(cut off) |
| 12.93 | 0.628 (0.580-0.676) | 0.637 (0.589-0.685) | 0.520 |
| 13.45 | 0.674 (0.626-0.722) | 0.584 (0.536-0.632) | 0.529 |

*AUC=0.655 (95% CI: 0.560-0.750), *p* =0.003.

Receiver operating characteristic, ROC;DOC, Distance on curve equaling square root of (1-Sen)^2^+ (1-Spe)^2^; AUC, Area under the ROC curve. LTL, leukocyte telomere length.

Supplenment Table 2. Risk factors predicted by the cut-off value of LTL in the study population.

|  | Total (n = 156) | Long LTL  (n = 100) | Short-LTL  (n = 56) | P Value |
| --- | --- | --- | --- | --- |
| Age, years | 65.29 ± 8.41 | 64.09 ± 8.68 | 67.45 ± 7.51 | 0.016 |
| Male, n (%) | 105 (67.3) | 71 (71.0) | 34 (60.7) | 0.189 |
| Smoker, n (%) | 89 (57.1) | 58 (58.0) | 31 (55.4) | 0.749 |
| CHD family history, n(%) | 90 (57.7) | 59 (59.0) | 31 (55.4) | 0.659 |
| Hypertension, n (%) | 83 (53.2) | 52 (52.0) | 31 (55.4) | 0.687 |
| Diabetes, n (%) | 48 (30.8) | 25 (25.0) | 23 (41.1) | 0.037 |
| Hyperlipidemia, n (%) | 56 (35.9) | 40 (40.0) | 16 (28.6) | 0.153 |
| SBP, mmHg | 134.31 ± 16.68 | 134.78 ± 15.84 | 133.48 ± 18.20 | 0.643 |
| DBP, mmHg | 79.99 ± 9.76 | 80.62 ± 10.15 | 78.86 ± 9.02 | 0.281 |
| TC, mmol/L | 4.13 ± 1.10 | 4.02 ± 1.17 | 4.33 ± 0.95 | 0.010 |
| TG, mmol/L | 1.62 ± 1.02 | 1.53 ± 0.90 | 1.76 ± 1.20 | 0.180 |
| LDL-C, mmol/L | 2.75 ± 1.04 | 2.70 ± 1.08 | 2.85 ± 0.98 | 0.369 |
| HDL-C, mmol/L | 1.11 ± 0.57 | 1.10 ± 0.55 | 1.15 ± 0.59 | 0.595 |
| Glucose, mmol/L | 6.21 ± 2.22 | 6.31 ± 2.35 | 6.02 ± 1.98 | 0.441 |
| HbA1c,(%) | 5.97 ± 1.00 | 6.01 ± 0.98 | 5.89 ± 1.03 | 0.499 |
| eGFR | 76.714 ± 19.03 | 78.56 ± 20.02 | 73.41 ± 16.77 | 0.105 |
| Treatment at discharge |  |  |  |  |
| Anti-plate, n (%) | 143 (91.7) | 94 (94.0) | 49 (87.5) | 0.159 |
| Statin, n (%) | 145 (92.9) | 92 (92.0) | 53 (94.6) | 0.536 |
| β-blockers, n (%) | 39 (25.0) | 22 (22.0) | 17 (30.4) | 0.248 |
| ACEI/ARB, n (%) | 72 (46.2) | 50 (50.0) | 22 (39.3) | 0.198 |
| CCB, n (%) | 50 (32.1) | 32 (32.0) | 18 (32.1) | 0.985 |
| Insulin, n (%) | 20 (12.8) | 9 (9.0) | 11 (19.6) | 0.056 |
| LTL(kb) | 13.51 (11.07, 15.45) | 14.86 (13.64, 16.97) | 10.59 (9.42, 11.47) | <0.001 |

Values were represented by mean ± SD or median and quartile (25%, 75%) and n (%)

CHD, coronary heart disease; SBP, systolic blood pressure; DBP, diastolic blood pressure; TC, total cholesterol; TG, triacylglycerol; LDL-C, low-density lipoproteins-cholesterol; HDL-C, high-density lipoproteins-cholesterol; HbA1c, glycated hemoglobin; eGFR, estimated glomerular filtration rate; ACEI, angiotensin-converting enzyme inhibitors; ARB, angiotensin receptor blockers; CCB, calcium channel blocker; LTL, leukocyte telomere length.

Table 3 Risk factors predicted by the cut-off value of LTL in the study population.

|  | Total(n = 156) | Long LTL(n = 100) | Short LTL(n = 56) | P Value |
| --- | --- | --- | --- | --- |
| Vessel location |  |  |  | 0.760 |
| LAD, n (%) | 100 (64.1) | 62 (62.0) | 38 (67.9) |  |
| RCA, n (%) | 43 (27.6) | 29 (29.0) | 14 (25.0) |  |
| LCX, n (%) | 13 (8.3) | 9 (9.0) | 4 (7.1) |  |
| RVD,(mm) | 3.31 ± 0.88 | 3.43 ± 0.90 | 3.10 ± 0.81 | 0.154 |
| MLD, (mm) | 1.40 ± 0.66 | 1.46 ± 0.69 | 1.30 ± 0.60 | 0.023 |
| DS, (%) | 59.26 ± 11.26 | 59.15 ± 11.65 | 59.45 ± 10.62 | 0.873 |
| Lesion length, (mm) | 20.70 (13.6, 28.4) | 20.1 (13.7, 27.4) | 21.8 (13.2, 29.5) | 0.380 |
| **Qualitative OCT analysis** | |  |  |  |
| TCFA, n (%) | 43 (27.6) | 17 (17.0) | 26 (46.4) | <0.001 |
| Macrophages, n (%) | 75 (48.1) | 31 (31.0) | 44 (78.6) | <0.001 |
| Microvessels, n (%) | 58 (37.2) | 28 (28.0) | 30 (53.6) | 0.002 |
| ChCs, n (%) | 76 (48.7) | 43 (43.0) | 33 (58.9) | 0.056 |
| Calcium rich plaque, n (%) | 64 (41.0) | 37 (37.0) | 27 (48.2) | 0.172 |
| **Quantitative OCT analysis** | |  |  |  |
| Lipid length, (mm) | 14.9 (9.1, 22.9) | 13.9 (8.7, 23.0) | 17.2 (11.3, 22.2) | 0.527 |
| Mean lipid arc, (°) | 206.0 (132.5, 254.0) | 180.5 (98.8, 246.3) | 227.0 (172.5, 254.8) | 0.026 |
| Lipid index, (mm*°) | 3119.6 (1242.5, 5171.4) | 2951.5 (1203.3, 4757.6) | 3696.5 (2339.9, 5654.0) | 0.082 |
| FCT, (μm) | 72.0 (46.5, 103.5) | 94.5 (58.0, 127.0) | 58 (40.0,76.5) | < 0.001 |

Values were represented by mean ± SD or median and quartile (25%, 75%) and n (%).

Lipid index = lipid length * mean lipid arc °.

TCFA, thin-cap fibroatheroma; LAD, left anterior descending coronary artery; RCA, right coronary artery; LCX, left circumflex artery; RVD, reference vessel diameter; MLD, minimal lumen diameter; DS, diameter stenosis; ChCs, Cholesterol Crystals; FCT: fibrous cap thickness.
